# Blood Haemoglobin Concentration Is Directly and Independently Related with Pulse Wave Velocity, a Measure of Large Artery Stiffness

**DOI:** 10.3390/jcm12247623

**Published:** 2023-12-11

**Authors:** Manoj Kumar Choudhary, Heidi Bouquin, Jere Hytönen, Jenni K. Koskela, Onni Niemelä, Pasi I. Nevalainen, Jukka Mustonen, Ilkka Pörsti

**Affiliations:** 1 Faculty of Medicine and Health Technology, Tampere University, 33014 Tampere, Finland; manoj.choudhary@tuni.fi (M.K.C.); heidi.bouquin@tuni.fi (H.B.); jenni.k.koskela@tuni.fi (J.K.K.); jukka.mustonen@tuni.fi (J.M.); 2 Department of Internal Medicine, Tampere University Hospital, 33520 Tampere, Finland; pasi.nevalainen@pirha.fi; 3 Department of Laboratory Medicine and Medical Research Unit, Seinäjoki Central Hospital, 60220 Seinäjoki, Finland; onni.niemela@epshp.fi

**Keywords:** artery stiffness, blood pressure, haemodynamics, haemoglobin, pulse wave velocity

## Abstract

High haemoglobin level has been associated with metabolic syndrome, elevated blood pressure (BP), and increased mortality risk. In this cross-sectional study, we investigated the association of blood haemoglobin with haemodynamics in 743 subjects, using whole-body impedance cardiography and pulse wave analysis. The participants were allocated to sex-stratified haemoglobin tertiles with mean values 135, 144, and 154 g/L, respectively. The mean age was similar in all tertiles, while body mass index was higher in the highest versus the lowest haemoglobin tertile. The highest haemoglobin tertile had the highest erythrocyte and leukocyte counts, plasma C-reactive protein, uric acid, renin activity, and aldosterone. The lipid profile was less favourable and insulin sensitivity lower in the highest versus the lowest haemoglobin tertile. Aortic BP, cardiac output, and systemic vascular resistance were similar in all tertiles, while the pulse wave velocity (PWV) was higher in the highest versus the lowest haemoglobin tertile. In linear regression analysis, age (Beta 0.478), mean aortic BP (Beta 0.178), uric acid (Beta 0.150), heart rate (Beta 0.148), and aldosterone-to-renin ratio (Beta 0.123) had the strongest associations with PWV (*p* < 0.001 for all). Additionally, haemoglobin concentration was an explanatory factory for PWV (Beta 0.070, *p* = 0.028). To conclude, blood haemoglobin concentration had a small direct and independent association with a measure of large artery stiffness.

## 1. Introduction

Cardiovascular diseases (CVD) due to atherosclerosis and its complications are the primary cause of mortality worldwide, representing 32% of all deaths [1]. In Europe as a whole, CVD accounts for over 3.9 million deaths annually (45% of all deaths) and 37% of all deaths in the European Union [2]. Although disability-adjusted life years (DALYs) due to CVD have decreased in most European countries over the last decade, CVD still accounts for the loss of more than 64 million DALYs in Europe, representing 23% of all DALYs lost [2].

A large study comprising 101,377 healthy blood donors reported that haemoglobin level was directly related with the level of systolic and diastolic BP [3]. Tapio et al. recently reported 20-year follow-up results from 967 subjects, showing that haemoglobin levels at the baseline were directly associated with systolic and diastolic BP, while high haemoglobin was also linked with less favourable prognosis [4]. In a study of 42 haemodialysis patients, erythropoietin administration was not only associated with an elevation of haemoglobin but also with a rise in BP [5], while in patients with orthostatic hypotension, erythropoietin treatment elevated BP while standing [6]. Increased haemoglobin concentration often accompanies insulin resistance and adverse metabolic profile [7,8]. Indeed, in subjects with high haemoglobin level the prevalence of the metabolic syndrome is high which increases their risk for CVD mortality [4]. Recently, both high and low haemoglobin concentrations were identified as independent predictors of cardiovascular events and all-cause mortality [4,9,10,11,12,13,14].

Increased pulse wave velocity (PWV) that designates large artery stiffness is a strong predictor of CVD and mortality, independent of the level of BP [15]. According to the Moens-Korteweg equation [16], PWV is proportional to the square root of the arterial wall elastic properties multiplied by the wall thickness divided by the artery diameter and the density of blood. Thus, pulse wave propagates faster in the arterial system during reduced arterial elasticity and increased blood density [16]. However, the Moens-Korteweg equation is based on simplifying assumptions and does not consider the dynamic elastic modulus and viscosity of arteries [16]. Xiao et al. reported that left ventricular ejection time influenced PWV, while PWV was also moderately modulated by peripheral arterial resistance [16]. Whole blood viscosity has been implicated as an independent risk factor for CVD [17,18,19] and peripheral vascular disease [20]. Blood viscosity is also an important determinant of local flow characteristics, which exhibits shear thinning behaviour, i.e., an exponential decrease with increasing shear rates. Both haematocrit and plasma properties influence blood viscosity [21]. The correlation between haemoglobin concentration and blood viscosity has been reported to be ~0.55 (*p* < 0.001), while haematocrit has been found as the single most important factor of whole blood viscosity [22].

To our knowledge, the association of haemoglobin with haemodynamic variables has not been extensively examined. The mechanisms underlying the relationship between haemoglobin concentration and cardiovascular events remain unclear, especially for high haemoglobin concentration and CVD. Due to the association of increased haemoglobin levels and incident metabolic syndrome [4,23] and the relationship of metabolic syndrome with artery stiffness even in absence of hypertension [24], the primary objective in this cross-sectional study was to examine the association of haemoglobin levels with large artery stiffness. We additionally investigated whether haemoglobin levels were related to BP level, wave reflection, cardiac output, or systemic vascular resistance.

## 2. Materials and Methods

### 2.1. Participants

All participants were from an ongoing study (DYNAMIC study; ClinicalTrials.gov identifier NCT01742702) that aimed to examine haemodynamics in primary and secondary hypertension compared to normotensive controls. The participant recruitment has been previously published [25] and altogether 743 subjects from 1349 were included. The study’s exclusion criteria included individuals with a medical history of (1) coronary artery disease, (2) stroke, (3) heart failure, (4) valvular heart disease, (5) diabetes, (6) chronic kidney disease, (7) secondary hypertension, (8) alcohol or substance abuse, (9) psychiatric illnesses, or (10) heart rhythm other than sinus rhythm.

A medical doctor conducted physical examinations and office BP measurements. Additionally, routine laboratory analyses for elevated BP were executed in all enrolled participants, following the guidelines of the European Society of Hypertension [26]. In addition to medical history, lifestyle habits and the use of dietary supplements, medicines, and other substances not registered as drugs were documented, including details about smoking habits and alcohol consumption measured in standard drinks (~12 g of absolute alcohol) per week.

The study included 394 men and 349 women, aged 19–80 years. In seated office measurements on a single occasion, 293 of the participants had BP < 140/90 mmHg, while 440 had BP ≥ 140/90 mmHg. Thus, 40% of the participants were in the normotensive range and 60% were in the hypertensive range of office BP measurements [26]. The subjects were divided into sex-adjusted haemoglobin tertiles (Tertile 1, *n* = 253; Tertile 2, *n* = 235; Tertile 3, *n* = 255).

Altogether, 397 (53.4%) of the participants used medications. 90 participants were on BP lowering agents. 80 females took systemic oestrogen, progestin, or their combination (contraception, hormone replacement therapy), and one used tibolone. 48 subjects were treated with statins, 21 with inhaled corticosteroids, 18 with selective serotonin or serotonin–norepinephrine reuptake inhibitors, and 30 were on a stable dose of thyroid hormone in an euthyroid state. Other medications in use were amitriptyline (*n* = 7), low dose acetylsalicylic acid (*n* = 26), allopurinol (*n* = 4), and warfarin (*n* = 5).

The study complies with the declaration of Helsinki and was approved by the ethics committee of the Tampere University Hospital (study code R06086M) and the Finnish Medicines Agency (Eudra-CT registration number 2006-002065-39). Signed informed consent was obtained prior to participation.

### 2.2. Laboratory Analyses

Blood and urine samples were collected from participants after about 12 h of fasting. Plasma concentrations of total cholesterol, high density lipoprotein-cholesterol (HDL-C), low density lipoprotein-cholesterol (LDL-C), triglycerides, C-reactive protein (CRP), sodium, potassium, glucose, cystatin C, and creatinine concentrations were determined using Cobas Integra 700/800 (F. Hoffmann-Laroche Ltd., Basel, Switzerland) or Cobas6000, module c501 (Roche Diagnostics, Basel, Switzerland). Insulin levels were determined using electrochemiluminescence immunoassay (Cobas e411, Roche Diagnostics) and blood cell counts were obtained using either the ADVIA 120 or 2120 analyzers (Bayer Health Care, Tarrytown, NY, USA). Overt renal disease in the participants was excluded by the results of the cystatin C and creatinine determinations and by the outcome of urine dipstick analysis with an automated refractometer test (Siemens Clinitec Atlas or Advantus, Siemens Healthcare GmbH, Erlangen, Germany). Quantitative insulin sensitivity check index (QUICKI) was calculated for the evaluation of insulin sensitivity [27] and glomerular filtration rate (eGFR) was estimated using the CKD-EPI cystatin C formula [28]. Plasma renin activity (PRA) and aldosterone concentration were analysed using radioimmunoassay (Plasma Renin Activity 125-I RIA Kit, DiaSorin, Saluggia, Italy; and Active Aldosterone RIA, Beckman Coulter, Fullerton, CA, USA). In this study, a positive screening result for primary aldosteronism was evaluated as presence of hypertension (BP > 140/90 mmHg) plus serum aldosterone >550 pmol/L and aldosterone to renin ratio >750 pmol/µg of angiotensin I/h [29,30].

### 2.3. Pulse Wave Analysis

Continuous pulse wave and radial BP were captured using an automated tonometric sensor (Colin BP-508T, Colin Medical Instruments Corp., San Antonio, TX, USA) that was attached to the left radial artery pulsation pulse with a wristband [25]. The radial BP signal was calibrated twice during each 5 min period by right brachial BP measurements. Aortic BP was derived using the SphygmoCor system (SphygmoCor PWMx^®^, AtCor medical, Sydney, Australia) [31]. Additionally, augmentation index (AIx, augmented pressure/pulse pressure*100), and AIx adjusted to heart rate 75/min (AIx@75) were determined [32].

### 2.4. Whole-Body Impedance Cardiography

Beat-to-beat heart rate, stroke volume, cardiac output, extracellular water, and PWV were recorded using whole-body impedance cardiography (CircMon^®^, JR Medical Ltd., Tallinn, Estonia). This technique detects changes in body electrical impedance during cardiac cycles and the configuration of electrodes has been previously reported [33,34]. Systemic vascular resistance was calculated using tonometric BP and evaluated cardiac index: normal central venous pressure (4 mmHg) was subtracted from mean arterial pressure and the value was divided by cardiac output. Systemic vascular resistance and cardiac output were related to body surface area and presented as indexes: cardiac index and systemic vascular resistance index (SVRI). The stroke volume values measured using CircMon^®^ correlate well with 3 dimensional ultrasound [35] and the cardiac output values correlate well with the values measured using thermodilution and direct oxygen Fick methods [33]. The whole-body impedance cardiography tends to overestimate PWV and a validated equation was utilized to calculate values corresponding to the ultrasound method (PWV = PWV_impedance_ × 0.696 + 0.864) [36]. By the use of this equation, the PWV values recorded using CircMon^®^ show good correlations with the values measured using the tonometric SphygmoCor^®^ method (r = 0.82, bias 0.02 m/s, 95% confidence interval −0.21 to 0.25) [32] or ultrasound (r = 0.91) [36].

### 2.5. Experimental Protocol

Haemodynamics were recorded in a quiet, temperature-controlled laboratory by research nurses. Participants were instructed to avoid smoking, caffeine-containing products, and heavy meals for at least 4 h, and alcohol consumption for more than 24 h prior to the recordings. The subjects rested supine, the left arm with the tonometric sensor abducted to 90 degrees in an arm support. After a 10 min period of acclimatization in the laboratory, the haemodynamic measurements were recorded continuously for 5 minutes. For the statistical analyses, the mean values of each 1 min period of recording were calculated. The measurement protocol has previously demonstrated good repeatability and reproducibility [34].

### 2.6. Statistics

Continuous variables were expressed as mean, standard error of the mean or standard deviation (SD), or as median [25th–75th percentile] as indicated. The baseline characteristics were depicted as sex-adjusted tertiles of haemoglobin (Table 1 and Table 2). The demographic and laboratory data were analysed using the analysis of variance (ANOVA) or the Kruskal-Wallis test, as appropriate. The homogeneity of variances was tested with the Levene’s test. Tertile proportions were compared using the chi square test.

The haemodynamics in the tertiles during the 5 min recordings were examined using generalized estimating equations (GEE). A linear scale response was applied and the autoregressive option for the correlation matrix was chosen because successive recordings of haemodynamic variables are autocorrelated. The statistics were adjusted for differences in body mass index (BMI) and plasma renin concentration by including these variables as covariates in the analyses (plasma renin concentration was the only significantly different laboratory variable between the tertiles after adjustment for BMI). The PWV analyses were additionally adjusted for mean aortic BP according to the guidelines [37]. The Bonferroni correction was applied in all post-hoc analyses.

Spearman’s correlations (r_S_) were calculated and variables that correlated with a significance level of *p* < 0.1 were included in the regression analyses. To evaluate the associations between PWV and haemoglobin, a multiple regression analysis with stepwise elimination was applied. The unstandardized coefficient B, standardized coefficient Beta, and R squared values of the regression analysis are presented in Table 3, and *p* < 0.05 was considered statistically significant. SPSS version 28.0 (IBM SPSS Statistics, Armonk, NY, USA) was used for the statistics.

## 3. Results

### 3.1. Study Population and Laboratory Values

Altogether, 394 (53%) male and 349 (47%) female subjects were included in the analyses (Table 1). The age range was 19–80 years. In the sex-adjusted tertiles of haemoglobin level, age, use of BP-lowering medications, height, extracellular water volume, smoking status, alcohol intake, seated office systolic BP, and supine systolic and diastolic BP measured by nurses were not different. Furthermore, the use of different classes of antihypertensive medications was very similar in the tertiles, the only difference being a lower prevalence of beta-blocker users in Tertile 2 versus Tertile 1. Whether evaluated by the proportions of those using antihypertensive medications in (i) office BP measurements using the cut-off level 140/90 mmHg, (ii) radial tonometric measurements in the laboratory using the cut-off level 135/85 mmHg, or (iii) aortic tonometric measurements using the cut-off level 125/85 mmHg, the prevalence of hypertensive individuals did not differ between the tertiles (the respective *p*-values were 0.134, 0.675, and 0.666) [26,38,39,40]. Weight, BMI, and seated office diastolic BP were higher in the highest versus lowest haemoglobin tertile (Table 1).

The average haemoglobin in the tertiles ranged (mean ± SD) from 135 ± 9 (Tertile 1) to 154 ± 9 (Tertile 3) (Table 2). In addition to haemoglobin and haematocrit, erythrocyte count was higher in Tertiles 2 and 3 than in Tertile 1. Mean corpuscular haemoglobin, leukocytes, CRP, uric acid, renin, and aldosterone were higher in Tertile 3 than in Tertiles 1 and 2. The aldosterone to renin ratio (*p* = 0.712) and the proportions of those with a positive screening result for primary aldosteronism (*p* = 0.180) did not differ between the tertiles. Plasma ferritin, triglycerides, LDL-C, and glucose concentrations were higher, while HDL-C and insulin sensitivity as judged by QUICKI were lower in Tertile 3 than in Tertile 1 (Table 2).

### 3.2. Haemodynamic Variables in the Tertiles of Haemoglobin Adjusted for Sex

**Figure 1:**
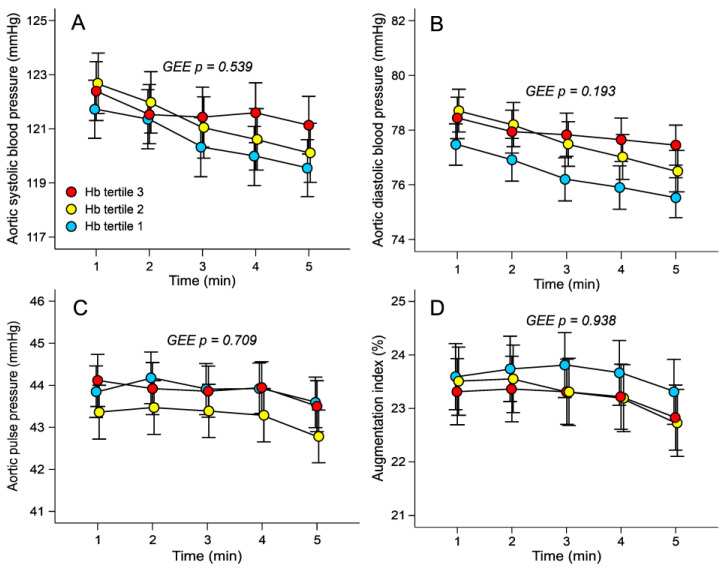
Line graphs show aortic systolic (**A**) and diastolic (**B**) blood pressure, aortic pulse pressure (**C**) and augmentation index (**D**) in sex-specific tertiles of blood haemoglobin concentration during 5-minute recordings in the supine position. Tertile 1 (*n* = 253), Tertile 2 (*n* = 235), and Tertile 3 (*n* = 255); mean ± standard error of the mean.

**Figure 2:**
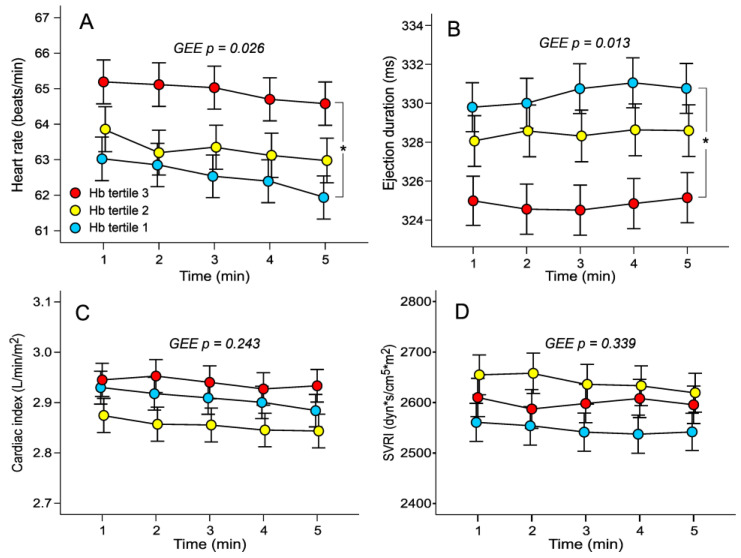
Line graphs show heart rate (**A**), ejection duration (**B**), cardiac index (**C**), and systemic vascular resistance index (**D**) in sex-specific tertiles of blood haemoglobin concentration during 5-min recordings; mean ± standard error of the mean; statistics by generalized estimating equations (GEE) adjusted for BMI, and plasma renin concentration; * *p* < 0.05.

**Figure 3:**
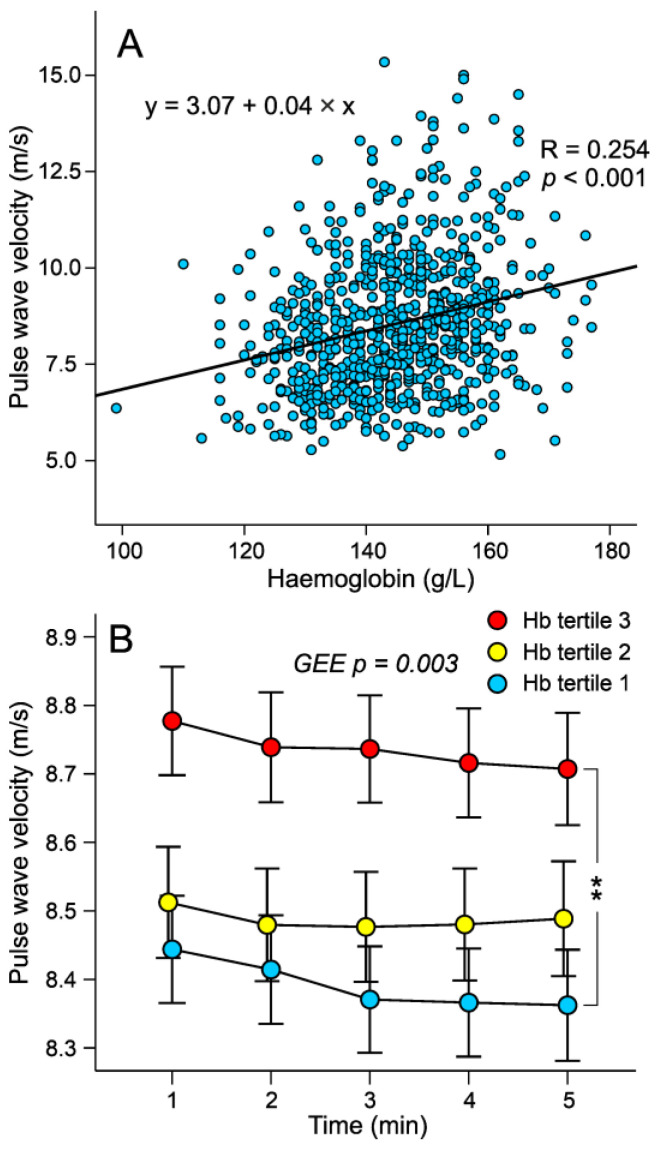
Scatter plot of the relationship between haemoglobin concentration and pulse wave velocity (**A**), and pulse wave velocity in sex-specific tertiles of blood haemoglobin concentration during 5-minute recordings (**B**); mean ± standard error of the mean; statistics by generalized estimating equations (GEE) adjusted for BMI, and plasma renin concentration; ** *p* < 0.01.

In analyses performed using the general linear model approach (GEE), aortic systolic and diastolic BP, aortic pulse pressure, and AIx were not different between the tertiles (Figure 1A–D). AIx@75 was also similar in the tertiles (23.6 ± 9.6, 23.3 ± 9.5, and 23.2 ± 9.6%, respectively). The GEE results indicated that heart rate was higher, whilst ejection duration was shorter, in the highest versus the lowest haemoglobin tertile (Figure 2A,B). No significant differences in stroke index were found between the tertiles (47 ± 7, 45 ± 7, and 46 ± 7 mL/m^2^, respectively). Cardiac index and SVRI did not differ between the tertiles (Figure 2C,D). A scatter plot based on raw data without any adjustments in Figure 3A shows that as the blood haemoglobin concentration increases, the propagation speed of the pulse wave also increases (r = 0.254, *p* < 0.001). In GEE analyses, aortic to popliteal PWV was higher in the highest versus the lowest haemoglobin tertile (Figure 3B). Neither AIx (r = 0.026, *p* = 0.482) nor AIx@75 (r = 0.062, *p* = 0.093) significantly correlated with the haemoglobin concentration.

### 3.3. Factor Influencing Pulse Wave Velocity in Stepwise Linear Regression Analysis

To examine the relationship of PWV with demographic, laboratory, and haemodynamic variables, we performed linear regression analyses (Table 3). In these analyses, age, mean aortic pressure, uric acid, heart rate, aldosterone to renin ratio, mean ejection duration, and triglycerides were the most significant explanatory factors for PWV. In addition, smoking status, haemoglobin, and vitamin 25(OH)D_3_ were minor or moderate but statistically significant explanatory factors for PWV (Table 3). When those 38 participants with a positive screening result for primary aldosteronism were excluded from the analysis, haemoglobin remained a modest independent explanatory factor for PWV (Beta 0.067, *p* = 0.041).

## 4. Discussion

The present results demonstrate a small independent association between haemoglobin concentration and PWV, a measure of large artery stiffness. While some reports suggest that high and low concentrations of haemoglobin are independent predictors of cardiovascular events [4,9,10,11,12,13,14] and that systolic and diastolic BP are directly associated with haemoglobin level [3,4], there is a dearth of information about the associations of blood haemoglobin levels with haemodynamic variables. Here we examined the association of haemoglobin with functional cardiovascular variables using non-invasive recordings of haemodynamics.

Age and BP level are major determinants of PWV [37]. Some previous reports show that high haemoglobin is moderately related with higher carotid-femoral or brachial-ankle PWV [41,42,43]. Sun et al. found that a high haemoglobin concentration was also related with a higher level of AIx@75 [43]. In addition, low haemoglobin level was related with lower brachial-ankle PWV among 69-year-old Japanese women [42]. However, the above studies did not examine the associations of haemoglobin levels with other haemodynamic variables. In the present study, the highest tertile with a mean haemoglobin concentration of 154 g/L presented with the highest PWV, while no differences in BP, wave reflection, cardiac output, and systemic vascular resistance were found between the tertiles. The lack of correlation between haemoglobin and AIx or AIx@75 can probably be explained by the fact that these measures of wave reflection are also influenced by aortic dimensions, heart rate, stroke volume, and systemic vascular resistance in addition to large artery stiffness [32,44,45]. In the regression analyses, age, prevailing BP, heart rate, uric acid, and aldosterone to renin ratio showed the strongest associations with PWV, corresponding to previous studies [25,46]. The impact of haemoglobin concentration was of a lower magnitude (Beta value = 0.07) in regression analyses, but it was still an independent explanatory factor for PWV.

Heart rate can directly influence PWV: small increases in heart rate slightly elevate carotid-to-femoral PWV, while greater increases can induce a clinically significant increase on carotid-to-femoral PWV [47]. Elevated resting heart rate is also an independent risk factor for cardiovascular events and future development of hypertension [48,49]. In the present study, the highest haemoglobin tertile had the highest heart rate and the shortest ejection duration. An inverse relation between haemoglobin level and left ventricular ejection duration has been previously reported [50,51], while an increase in haemoglobin over time is associated with a decrease in left ventricular ejection duration [51,52]. Under resting conditions, PWV was inversely correlated with left ventricular ejection time in 102 young healthy males [53], and among 1107 adult male and 1913 adult female subjects [54]. In our regression model, PWV was directly related with heart rate (Beta 0.148) and inversely related with left ventricular ejection duration (Beta = −0.100), corresponding to the above findings.

Several possible mechanisms could link increased haemoglobin levels with higher PWV. Haemoglobin and red cell count are independently related with insulin resistance even in healthy persons [7,8,55]. Insulin resistance, in turn, has been independently linked with PWV in 455 normoglycemic normotensive postmenopausal women [56], and in 1541 nondiabetic subjects without inflammation, malignant diseases, autoimmune disorders, renal impairment, or abnormal hepatic function tests [57]. Tapio et al. reported that high haemoglobin levels in middle-aged subjects were associated with adiposity, elevated heart rate, hypertension, glucose intolerance, dyslipidaemia, and higher inflammatory burden [4]. In the present study, the highest haemoglobin tertile presented with higher BMI, diastolic office BP, and plasma concentrations of triglycerides, LDL-C, and glucose, and lower HDL-C and insulin sensitivity, than the lowest tertile. All of the above factors are features of insulin resistance and the metabolic syndrome and these conditions are also characterized by endothelial dysfunction [58]. Recently, metabolic syndrome was found to be characterized by simultaneous bone marrow activation and increased haematopoiesis [14]. This view aligns with the current findings showing a gradual rise in red blood cell count along increasing levels of haemoglobin and haematocrit. Additionally, blood leukocyte count was higher in individuals within the highest versus the lowest haemoglobin tertile. These results are compatible with bone marrow activation in individuals with the highest haemoglobin levels [14], who also exhibited several characteristics of the metabolic syndrome.

Blood viscosity influences PWV [59,60], while haemoglobin and haematocrit are the most significant determinants of blood viscosity [22]. Plasma viscosity also influences blood viscosity, while elevated plasma and whole blood viscosity have both been reported in hypertensive subjects [60,61]. The physiological compensation of viscosity-related decrease in blood flow is an increase in pressure or peripheral vasodilation, while decreased viscosity is related with increased cardiac output, and increased viscosity is related with decreased cardiac output [62]. Blood viscosity is also a modulator of endothelial nitric oxide synthesis via its effect on wall shear stress, whereby an increase in blood viscosity should promote vasodilation [21]. However, this mechanism is impaired during insulin resistance, which is characterized by deficient endothelium-mediated control of arterial tone [58]. Furthermore, haemoglobin is a scavenger of nitric oxide, whereby increased levels of free haemoglobin may promote vasoconstriction by binding nitric oxide [63]. In the present study, blood haemoglobin was a minor but statistically significant explanatory factor for PWV. Whether higher PWV during higher haemoglobin concentration reflects less compliant large arteries, or merely an effect of higher blood viscosity on the propagation of the pressure wave, the subsequent stress upon the target organs caused by the systolic pressure wave is increased in either case. Of note, higher blood viscosity has been linked with a higher incidence of cardiovascular events in the Edinburgh Artery Study during 5 years of follow-up [18].

The current study has limitations and the results should be interpreted cautiously. The present methods have been validated against invasive measurements, three-dimensional ultrasound, and tonometric recordings of PWV [31,32,33,35]. Nevertheless, the non-invasive evaluations of stroke volume and cardiac output are based on mathematical analysis of the bioimpedance signal that simplifies physiology [33]. Central BP was also mathematically derived from the radial artery tonometric signal [31]. The present recordings lasted for 5 min, which gives a rather narrow window of observation for the examination of haemodynamics. Yet, when compared with single measurements of BP and heart rate, the present analyses were based on recordings collected from more than 300 cardiac cycles. Thus, the average values of all haemodynamic variables during each minute of measurement represented a rather large number of individual values. Although some criticism regarding the reliability of the tonometric BP recording has been presented [64,65], we recently reported that the recorded BP values well corresponded to the level of ambulatory daytime BP among 410 participants [66]. While most of the present study subjects were devoid of medications with direct cardiovascular influences, also other medications used by the participants may have influenced the results. Importantly, the cross-sectional design does not allow conclusions about causality and the present findings should be confirmed in follow-up studies. Finally, elevated haemoglobin levels could potentially be linked to other coexisting conditions, such as the metabolic syndrome, which contribute to large artery stiffening and increase PWV values. In this case, the observed correlation between haemoglobin and PWV could rather be attributed to underlying health conditions than a direct causal relationship.

## 5. Conclusions

The present results showed that high haemoglobin concentration had a small direct and independent association with PWV, an acknowledged measure of large artery stiffness and CVD risk [15].

## Figures and Tables

**Table 1:** Demographics, clinical characteristics, and laboratory results in tertiles of plasma haemoglobin concentration adjusted for sex.

Demographics and Clinical Characteristics	Hb Tertile 1 (*n* = 253)	Hb Tertile 2 (*n* = 235)	Hb Tertile 3 (*n* = 255)
Males (%)	53.8	51.0	54.1
Age (years)	46.9 (12.9)	47.4 (11.5)	46.8 (11.6)
Antihypertensive medication in use (%)	14.6	8.5	12.9
Angiotensin-converting enzyme inhibitor (%)	5.5	3.4	4.3
Angiotensin receptor blocker (%)	4.7	3.0	4.3
Beta blocker (%)	7.9	2.6*	7.1
Beta and alpha blocker (%)	0.4	0.0	0.8
Calcium channel blocker (%)	6.7	3.4	4.7
Thiazide (%)	4.7	3.8	6.3
Furosemide (%)	0.8	0.0	0.8
Potassium-sparing diuretic (%)	1.6	0.4	1.2
Prazosin (%)	0.8	0.0	0.4
Minoxidil (%)	0.4	0.0	0.0
Height (cm)	173.3 (9.0)	173.3 (9.8)	173.5 (9.0)
Weight (kg)	79.9 (16.0)	81.6 (15.3)	84.5 (16.8) *
Body mass index (kg/m^2^)	26.5 (4.4)	27.1 (4.5)	27.9 (4.7) *
Extracellular water volume (L)	12.92 (2.02)	12.98 (1.88)	13.21 (1.78)
Current smokers (%)	10.7	11.5	16.5
Alcohol consumption (standard drinks/week)	3.0 [0.5–7.0]	2.0 [0.5–6.0]	3.0 [1.0–6.0]
Seated office blood pressure measured by physician			
Systolic (mmHg)	140 (21)	141 (20)	142 (19)
Diastolic (mmHg)	88 (12)	90 (12)	91 (11) *
Supine brachial blood pressure measured by nurse			
Systolic (mmHg)	133 (19)	133 (18)	134 (18)
Diastolic (mmHg)	80 (11)	81 (10)	83 (12)
Office blood pressure ≥ 140/90 mmHg ^†^ (%)	55.9	61.4	64.6
Radial blood pressure in the laboratory ≥ 135/85 mmHg ^†^ (%)	43.9	44.3	47.4
Aortic blood pressure in the laboratory ≥ 125/85 mmHg ^†^ (%)	39.1	39.6	42.7

Results shown as mean (standard deviation) or median [25th–75th percentile]. * *p* < 0.05 vs. Tertile 1. ^†^ Either systolic or diastolic blood pressure equal to or above the cut-off value.

**Table 2:** Fasting blood and plasma biochemistry results in tertiles of plasma haemoglobin concentration.

Blood and Plasma Biochemistry	Hb Tertile 1 (*n* = 253)	Hb Tertile 2 (*n* = 235)	Hb Tertile 3 (*n* = 255)
Haemoglobin (g/L)	135 (9)	144 (8) *	154 (9) *^†^
Haematocrit	0.40 (0.03)	0.42 (0.02) *	0.45 (0.03) *^†^
Mean corpuscular haemoglobin (pg)	30.1 (1.7)	30.4 (1.4)	30.8 (1.4) *^†^
Mean corpuscular volume (fl)	88.9 (4.6)	88.6 (3.7)	89.1 (4.2)
Erythrocyte count (×10^12^/L)	4.50 (0.32)	4.75/0.32) *	5.03 (0.38) *^†^
Thrombocyte count (×10^9^/L)	259 (55)	263 (255)	253 (56)
Leukocyte count (×10^9^/L)	5.61 (2.30)	5.76 (1.33)	6.33 (1.67) *^†^
Ferritin (µg/L)	51.5 [22.2–104.9]	60.5 [26.7–121.1]	73.5 [39.7–139.3] *
Sodium (mmol/L)	140.6 (2.1)	140.3 (2.2)	140.4 (2.1)
Potassium (mmol/L)	3.79 (0.29)	3.80 (0.30)	3.79 (0.31)
Creatinine (μmol/L)	74 (13)	75 (14)	75 (14)
Cystatin C (mg/L)	0.86 (0.17)	0.86 (0.16)	0.88 (0.16)
PTH (pmol/L)	4.6 (1.8)	4.8 (2.0)	4.7 (1.9)
Phosphate	0.97 (0.15)	0.95 (0.17)	0.96 (0.17)
Calcium (mmol/L)	2.31 (0.11)	2.31 (0.12)	2.32 (0.10)
25(OH)D_3_ (nmol/L)	74 (38)	71 (32)	73 (39)
1,25(OH)_2_D_3_ (pmol/L)	107 (33)	109 (36)	105 (30)
C-reactive protein (mg/L)	0.8 [0.5–1.9]	0.8 [0.5–1.9]	1.1 [0.5–2.2] *^†^
Uric acid (μmol/L)	305 (80)	305 (80)	325 (75) *^†^
Renin activity (ng Ang I/mL/h)	0.64 [0.38–1.23]	0.78 [0.4–1.37]	1.01 [0.54–1.79] *^†^
Aldosterone (pmol/L)	416 [301–532]	429 [323–595]	489 [373–686] *^†^
Aldosterone to renin ratio	733 (527)	696 (460)	697 (708)
Positive screening result for PA (n/%)	9/3.6%	11/4.7%	18/7.0%
Total cholesterol (mmol/L)	5.03 (1.11)	5.22 (0.98)	5.22 (1.01)
Triglycerides (mmol/L)	0.99 [0.68–1.42]	1.07 [0.81–1.55]	1.17 [0.82–1.75] *
HDL cholesterol (mmol/L)	1.60 (0.48)	1.57 (0.42)	1.49 (0.42) *
LDL cholesterol (mmol/L)	2.93 (1.05)	3.13 (0.89)	3.19 (0.93) *
Glucose (mmol/L)	5.5 (0.6)	5.5 (0.6)	5.6 (0.7) *
Insulin (mU/L)	7.9 (5.7)	10.5 (26.4)	10.5 (9.1)
QUICKI	0.359 (0.035)	0.353 (0.043)	0.348 (0.047) *

Results shown as mean (standard deviation) or median [25th–75th percentile]. Due to missing values, all blood and plasma biochemistry results were available in Tertile 1 for 232 subjects, in Tertile 2 for 212 subjects, and in Tertile 3 for 217 subjects. PTH, parathyroid hormone; 25(OH)D_3_, 25-hydroxyvitamin D_3_; 1,25(OH)_2_D_3_, 1,25-dihydroxyvitamin D_3_; PA, primary aldosteronism; HDL, high-density lipoprotein; LDL, low-density lipoprotein; QUICKI, quantitative insulin sensitivity check index. * *p* < 0.05 vs. Tertile 1; ^†^ *p* < 0.05 vs. Tertile 2.

**Table 3:** Significant explanatory variables for pulse wave velocity in linear regression analysis with stepwise elimination.

Pulse Wave Velocity, Model (R^2^ = 0.581)	B	Beta	*p*-Value
(constant)	1.097		
Age	0.074	0.478	<0.001
Mean aortic pressure	0.024	0.178	<0.001
Uric acid	0.003	0.150	<0.001
Mean heart rate	0.028	0.148	<0.001
Aldosterone to renin ratio	0.00041	0.123	<0.001
Mean ejection duration	−0.009	−0.100	0.008
Triglycerides	0.278	0.098	0.001
Smoking (present)	−0.396	−0.073	0.007
Haemoglobin	0.011	0.070	0.028
Vitamin 25(OH)D_3_	−0.003	−0.057	0.034

Other variables included in model: Sex, body mass index, smoking status, categorized use of alcohol, estimated cystatin C-based glomerular filtration rate [28], blood leukocyte count, plasma concentrations of C-reactive protein, low-density lipoprotein and high-density lipoprotein cholesterol, calcium, phosphate, parathyroid hormone, glucose, insulin, 1,25(OH)_2_D_3_, renin, aldosterone, and ferritin.

## Data Availability

Analyses and generated datasets that support the current study are not available publicly. The datasets are available from the corresponding author on reasonable request.
